# Supplementary material for: Functional systemic CD4 immunity is required for clinical responses to PD‐L1/PD‐1 blockade therapy
Source: EMBO Mol Med. 2019 Jun 6;11(7):e10293. doi: 10.15252/emmm.201910293 (PMC6609910; doi:10.15252/emmm.201910293)
Supplement: Supplementary file 1 — Appendix [file EMMM-11-e10293-s001.pdf]

APPENDIX INFORMATION

Functional systemic CD4 immunity is required for clinical responses to PD-L1/PD-1 blockade therapy

Miren Zuazo, Hugo Arasanz, Gonzalo Fernández-Hinojal, Maria Jesus García-Granda, María Gato, Ana Bocanegra, Maite Martínez, Berta Hernández, Lucía Teijeira, Idoia Morilla, Maria Jose Lecumberri, Angela Fernández de Lascoiti, Ruth Vera, Grazyna Kochan, and David Escors

APPENDIX MATERIAL

Appendix Figure S1

Appendix Figure S1

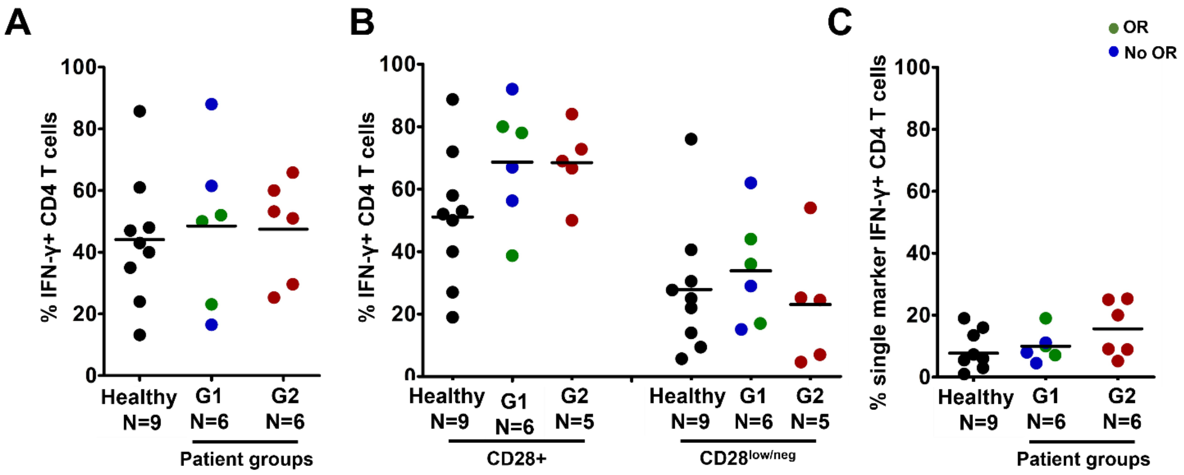

Appendix Figure S1. IFN responses in activated systemic CD4 T cells from NSCLC patients. (A) The dot plot graph shows the percentage of total IFN-γ-expressing cells in systemic CD4 T cells activated with anti-CD3/anti-CD28 antibodies. Data from individual age-matched healthy donors, G1 or G2 patients are shown. Most of these T cells express multiple cytokines apart from IFN-γ (Figure 4). N, number of patients; Blue, G1 non-responder patients; Green, responder patients; red, G2 patients. No statistical differences were found between the three groups. **(B)** as in (A) but separating $CD28^{+}$ (non-T_{HD}) from $CD28^{low/negative\ cells}$ (T_{HD}). No statistical differences were found between the groups. **(C)** as in (A) but with CD4 T cells expressing only IFN-γ, which represents single-expressing exhausted cells.
