# Supplementary material for: Functional systemic CD4 immunity is required for clinical responses to PD‐L1/PD‐1 blockade therapy
Source: EMBO Mol Med. 2019 Jun 6;11(7):e10293. doi: 10.15252/emmm.201910293 (PMC6609910; doi:10.15252/emmm.201910293)
Supplement: Supplementary file 2 — Expanded View Figures PDF [file EMMM-11-e10293-s002.pdf]

Expanded View Figures

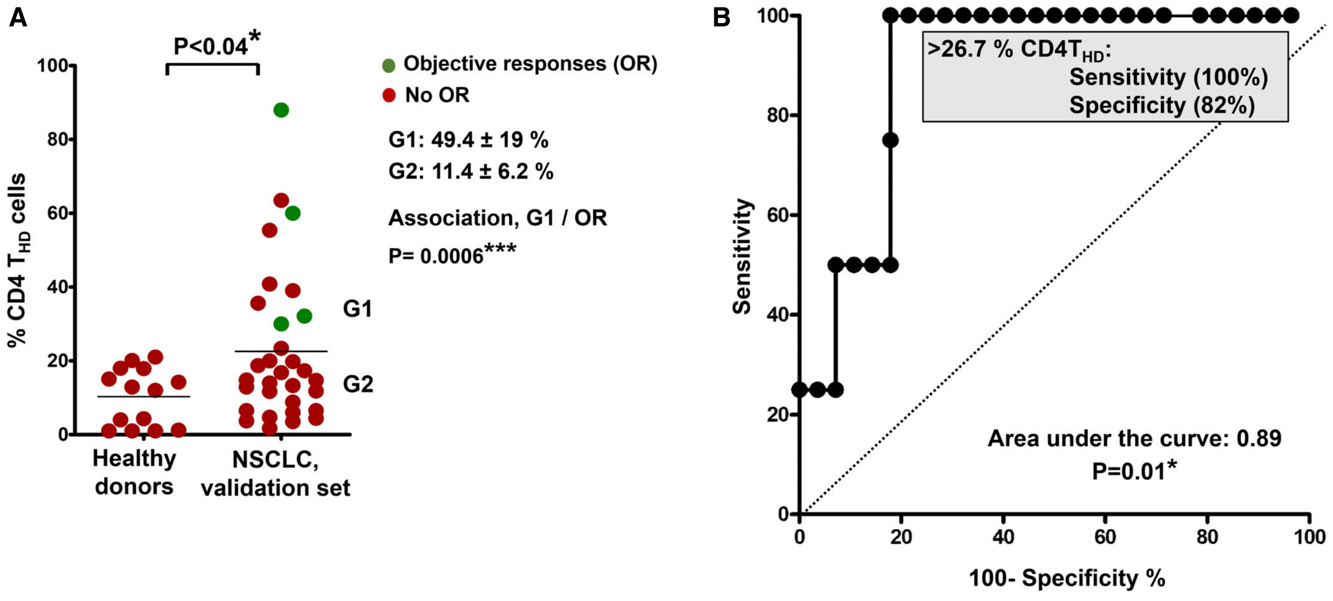

Figure EV1. Validation dataset.

A Distribution of circulating CD4 T_{HD} cells within $CD4^+$ $CD14^{negative}$ cells in healthy donors (N = 14) and in NSCLC patients constituting the validation set (N = 32). G1 and G2 groups are indicated and separated by the mean (horizontal line). The means ± standard deviations of CD4 T_{HD} cells in G1 and G2 groups are shown on the right, as well as the association between G1 profiles and objective responses by Fisher's test. Differences between healthy donors and NSCLC patients were tested with the Mann–Whitney U test.

B ROC analysis of CD4 T_{HD} quantification in the validation dataset and objective responses. The cut-off value for identification of responses is shown in the graph.

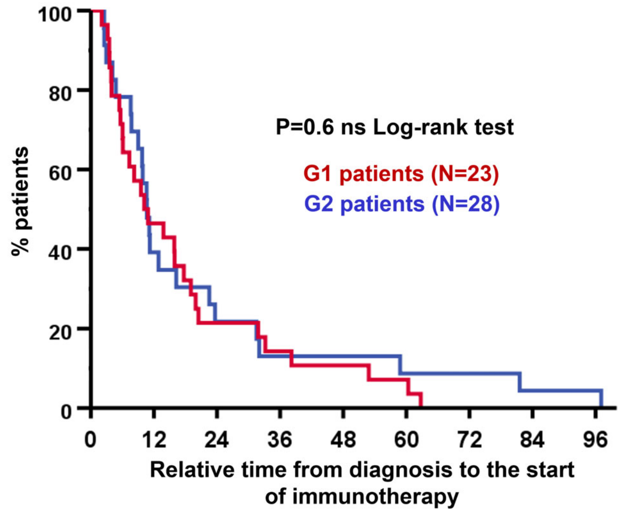

Figure EV2. CD4 T-cell profiling does not have significant prognostic value.

Kaplan–Meier plot of relative time elapsed from diagnosis to the start of immunotherapy for G1 (blue) and G2 patient cohorts (red), as indicated. No significant differences were found.

Source data are available online for this figure.

© 2019 The Authors

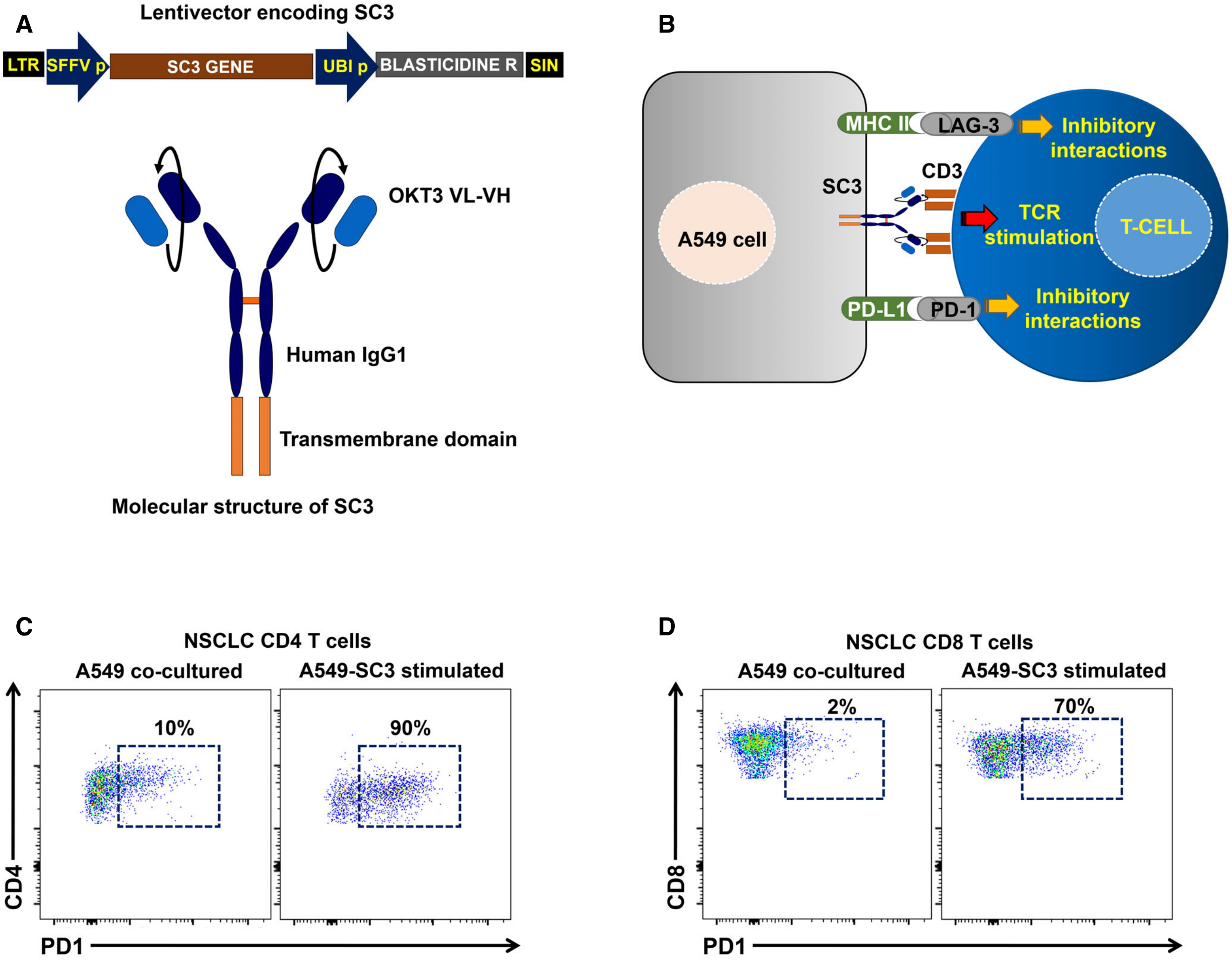

Figure EV3. Ex vivo human lung adenocarcinoma T-cell recognition system.

A Top, lentivector co-expressing an anti-CD3 single-chain antibody gene (SC3) and blasticidin resistance for selection. SFFVp, spleen focus-forming virus promoter; UBIp, human ubiquitin promoter; LTR, long terminal repeat; and SIN, U3-deleted LTR leading to a self-inactivating lentivector. Bottom, molecular structure of the SC3 molecule, which is anchored to the cell membrane by a transmembrane domain as indicated. OKT3 VL, variable region of the light chain from the anti-CD3 antibody OKT3; VH, variable region of the heavy chain from the anti-CD3 antibody OKT3.

B Scheme of the cell-to-cell interactions mediated by the lentivector-modified A549 cell and T cells including SC3/CD3, PD-L1/PD-1, and MHCII/LAG-3 interactions as indicated.

C, D Representative flow cytometry density plots with the upregulation of PD-1 expression in CD4 (C) and CD8 T cells (D) from NSCLC patients following co-incubation with A549-SC3 cell as indicated (right graph), or with unmodified A549 control (left graph). Percentages of PD-1^{+} T cells are shown within the graphs.

 © 2019 The Authors

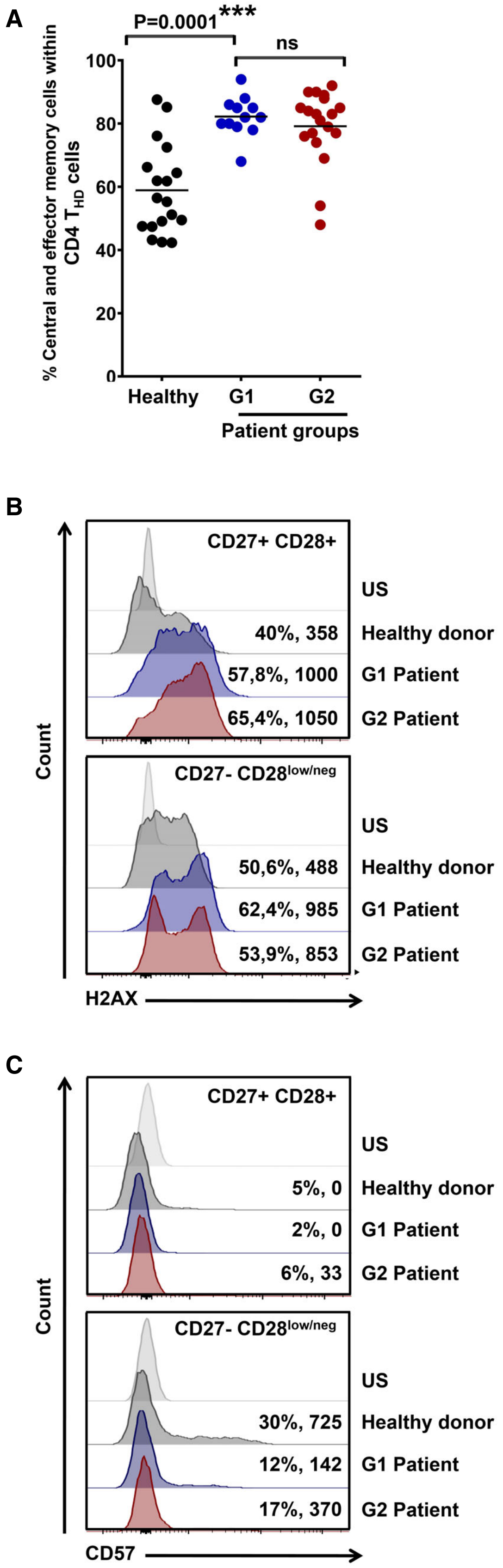

Figure EV4. CD4 T_{HD} cells in NSCLC patients are mainly non-senescent memory subsets.

A Scatter plot graphs of the percentage of memory phenotypes in baseline CD4 T_{HD} cells according to CD62L-CD45RA expression (% CD45RA^{negative} CD62L^{positive} central-memory + % CD45RA^{negative} CD62L^{negative} effector-memory cells) in a sample of healthy donors (n = 18), G1 (n = 12) and G2 (n = 19) patients. Relevant statistical comparisons are shown by one-way ANOVA followed by Tukey's test.

B, C Expression of the genotoxic damage makers H2AX (B) and CD57 (C) by flow cytometry in CD4 T-cell subsets from an aged-matched healthy donor, and NSCLC G1 and G2 patients as indicated. Percentage of positivity and mean fluorescent intensities are indicated for each population. Top, histogram analysis within CD27^{+} CD28^{+} CD4 T cells, and bottom, CD27^{negative} CD28$^{low/negative}$ counterparts as indicated. US, unstained control.

© 2019 The Authors

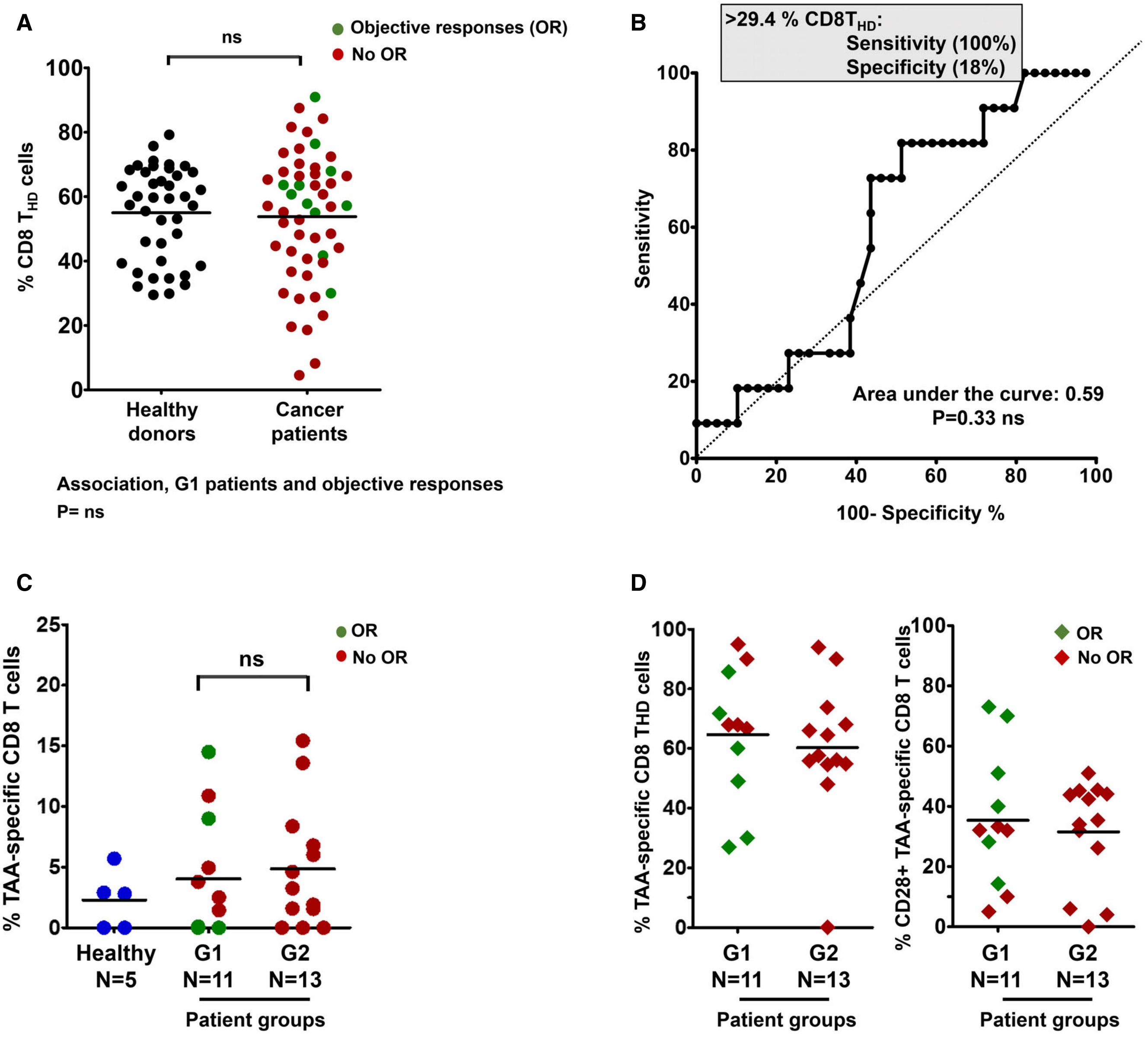

Figure EV5. Systemic CD8 responses in NSCLC patients.

- A Percentage of circulating highly differentiated CD8 cells in age-matched healthy donors (n = 40) and NSCLC patients (n = 51) before undergoing immunotherapies. Relevant statistical comparisons are shown by the test of Mann–Whitney. In green, objective responders (OR). In red, no OR.
- B ROC analysis of baseline CD8 T_{HD} quantification as a function of objective clinical responses.
- C Dot-plot of lung cancer antigen-specific CD8 T cells obtained before the start of immunotherapies and stimulated with A549-loaded autologous DCs autologous DC in healthy donors (n = 5), G1 (n = 11) and G2 (n = 13) patients, as indicated. Relevant statistical comparisons are indicated by the test of Kruskal–Wallis.
- D Left dot-plot, percentage of CD28-negative CD8 T cells within TAA-specific CD8 subsets in G1 (n = 11) and G2 (n = 13) patients, as indicated. Right dot-plot, same as left but with CD28-positive subsets. Green, objective responders (OR) and red, no ORs. N, number of biological replicates (independent patients).

© 2019 The Authors
