# Supplementary material for: Functional systemic CD4 immunity is required for clinical responses to PD‐L1/PD‐1 blockade therapy
Source: EMBO Mol Med. 2019 Jun 6;11(7):e10293. doi: 10.15252/emmm.201910293 (PMC6609910; doi:10.15252/emmm.201910293)
Supplement: Supplementary file 3 — Table EV1 [file EMMM-11-e10293-s003.docx]

**Table EV1. Baseline patient characteristics**

| Variable | All patients (N=51) |
| --- | --- |
| Sex |  |
| Female | 15 (29,4) |
| Male | 36 (70,6) |
| Age |  |
| <60 | 15 (29,4) |
| ≥60 | 36 (70,6) |
| Histology |  |
| Squamous | 12 (23,5) |
| Non- Squamous | 39 (76,5) |
| Inmunotherapy ttreatment |  |
| Pembrolizumab | 8 (15,7) |
| Nivolumab | 22 (43,1) |
| Atezolizumab | 21 (41,2) |
| PDL1 status |  |
| 0% | 16 (31,3) |
| 1-4% | 5 (9,8) |
| 5-49% | 10 (19,6) |
| ≥ 50% | 8 (15,7) |
| Undetermined | 12 (23,5) |
| Mutation status |  |
| No | 49 (96) |
| EGFR | 1 (2) |
| ROS1 | 1 (2) |
| Smoking status |  |
| Smoker | 46 (90,2) |
| Non-smoker | 5 (9,8) |
| Treatment line |  |
| 2nd | 34 (66,7) |
| 3th | 14 (27,5) |
| 4th or higher | 3 (5,9) |
| Previous systemic therapies (previous 3 months) |  |
| Platinium-based therapy | 19 (37,3) |
| Non-platinium based therapy | 17 (33,3) |
| No | 15 (29,4) |
| ECOG |  |
| 0-1 | 26 (51) |
| 2-4 | 14 (27,5) |
| Undetermined | 11 (21,5) |
| GRImScore |  |
| 0-1 | 26 (51) |
| 2-3 | 14 (27,5) |
| Undetermined | 11 (21,5) |
| Liver metastases |  |
| No | 38 (74,5) |
| Yes | 13 (25,5) |
| Number of sites involved |  |
| ≤2 | 15 (29,4) |
| ≥3 | 36 (70,6) |
| CD4 THD Profiling |  |
| G1 profile | 23 (45,1) |
| G2 profile | 28 (54,9) |
| Responses |  |
| Partial response | 11 (21,6) |
| Progression disease | 36 (70,6) |
| Stable disease | 4 (7,8) |
