# Supplementary material for: Functional systemic CD4 immunity is required for clinical responses to PD‐L1/PD‐1 blockade therapy
Source: EMBO Mol Med. 2019 Jun 6;11(7):e10293. doi: 10.15252/emmm.201910293 (PMC6609910; doi:10.15252/emmm.201910293)
Supplement: Supplementary file 4 — Table EV2 [file EMMM-11-e10293-s004.docx]

**Table EV2. Association of CD4 T cell profiles with GRIm score.**

|  | CD4 THD profiles | | Clinical responses | |
| --- | --- | --- | --- | --- |
| GRImScore | **G1** | **G2** | **Objective responders** | **Progressors** |
| 0-1 | 11 | 15 | 7 | 19 |
| 2-4 | 9 | 5 | 3 | 11 |
|  |  | | | |
| Association, P | 0.14 ns | | 0.2 ns | |
