# Supplementary material for: Functional systemic CD4 immunity is required for clinical responses to PD‐L1/PD‐1 blockade therapy
Source: EMBO Mol Med. 2019 Jun 6;11(7):e10293. doi: 10.15252/emmm.201910293 (PMC6609910; doi:10.15252/emmm.201910293)
Supplement: Supplementary file 5 — Source Data for Expanded View [file EMMM-11-e10293-s005.pdf]

**Source data from Figure EV2. Relative time from diagnosis to the start of immunotherapies**

| | | |
|---|---|---|
| G1 | 1 | 2,530 |
| | 2 | 2,563 |
| | 3 | 2,924 |
| | 4 | 4,107 |
| | 5 | 4,764 |
| | 6 | 7,589 |
| | 7 | 7,721 |
| | 8 | 9,002 |
| | 9 | 9,758 |
| | 10 | 9,856 |
| | 11 | 10,612 |
| | 12 | 10,645 |
| | 13 | 11,105 |
| | 14 | 11,203 |
| | 15 | 12,879 |
| | 16 | 16,230 |
| | 17 | 22,538 |
| | 18 | 23,622 |
| | 19 | 31,507 |
| | 20 | 32,033 |
| | 21 | 58,809 |
| | 22 | 81,544 |
| | 23 | 97,051 |
| G2 | 1 | 2,070 |
| | 2 | 3,220 |
| | 3 | 3,483 |
| | 4 | 3,548 |
| | 5 | 3,877 |
| | 6 | 3,943 |
| | 7 | 5,421 |
| | 8 | 5,651 |
| | 9 | 6,045 |
| | 10 | 6,078 |
| | 11 | 7,326 |
| | 12 | 8,246 |
| | 13 | 9,528 |
| | 14 | 10,152 |

| | | |
|---|---|---|
| | 15 | 10,875 |
| | 16 | 13,799 |
| | 17 | 15,869 |
| | 18 | 15,934 |
| | 19 | 17,676 |
| | 20 | 19,023 |
| | 21 | 19,943 |
| | 22 | 20,468 |
| | 23 | 31,836 |
| | 24 | 33,183 |
| | 25 | 38,144 |
| | 26 | 52,862 |
| | 27 | 60,353 |
| | 28 | 62,752 |
