# Supplementary material for: Functional systemic CD4 immunity is required for clinical responses to PD‐L1/PD‐1 blockade therapy
Source: EMBO Mol Med. 2019 Jun 6;11(7):e10293. doi: 10.15252/emmm.201910293 (PMC6609910; doi:10.15252/emmm.201910293)
Supplement: Supplementary file 7 — Source Data for Figure 1 [file EMMM-11-e10293-s006.pdf]

Progression Free Survival G1 vs G2 patients. Source data for the Kaplan-Meier plot from Figure 1

G1	1	3,143
	2	3,714
	3	4,000
	4	5,571
	5	6,143
	6	6,143
	7	6,286
	8	6,286
	9	9,143
	10	9,286
	11	10,857
	12	11,429
	13	23,143
	14	23,714
	15	32,429
	16	34,143
	17	35,143
	18	35,143
	19	36,143
	20	46,286
	21	53,143
	22	54,429
	23	63,429
G2	1	1,143
	2	1,571
	3	1,714
	4	3,714
	5	5,000
	6	5,571
	7	5,714
	8	5,714
	9	5,714
	10	5,857
	11	5,857
	12	6,000
	13	6,000
	14	6,143
	15	6,143

	16	6,286
	17	6,429
	18	6,429
	19	6,429
	20	6,571
	21	8,286
	22	8,857
	23	9,857
	24	10,857
	25	12,429
	26	13,429
	27	18,857
	28	35,857
