# Supplementary material for: Functional systemic CD4 immunity is required for clinical responses to PD‐L1/PD‐1 blockade therapy
Source: EMBO Mol Med. 2019 Jun 6;11(7):e10293. doi: 10.15252/emmm.201910293 (PMC6609910; doi:10.15252/emmm.201910293)
Supplement: Supplementary file 8 — Source Data for Figure 7 [file EMMM-11-e10293-s007.pdf]

Source data of Progression Free Survival from Figure 7A G1 + PD-L1 >/= 5% (only known PD-L1 status)

NO	1	1,571
	2	3,143
	3	3,714
	4	4,000
	5	5,000
	6	5,571
	7	5,714
	8	5,857
	9	5,857
	10	6,000
	11	6,143
	12	6,143
	13	6,286
	14	6,286
	15	6,286
	16	6,429
	17	6,429
	18	6,429
	19	6,571
	20	8,286
	21	9,857
	22	10,857
	23	12,000
	24	13,000
	25	14,000
	26	36,143
	27	36,429
YES	1	6,143
	2	9,143
	3	9,143
	4	10,857
	5	23,143
	6	33,000
	7	34,714
	8	35,714
	9	35,714
	10	46,857

	11	55,000
	12	64,000

Source data of Progression Free Survival from Figure 7B G1 + PD-L1 >/= 5 (vs the rest of patients)

No	1	1,143
	2	1,571
	3	1,714
	4	3,143
	5	3,714
	6	3,714
	7	4,000
	8	5,000
	9	5,571
	10	5,714
	11	5,714
	12	5,714
	13	5,857
	14	5,857
	15	6,000
	16	6,000
	17	6,143
	18	6,143
	19	6,286
	20	6,286
	21	6,286
	22	6,429
	23	6,429
	24	6,429
	25	6,571
	26	8,286
	27	8,857
	28	9,857
	29	10,857
	30	11,429
	31	12,429
	32	13,429
	33	18,857
	34	35,857
	35	36,143

Yes	1	6,143
	2	6,143
	3	9,143
	4	10,857
	5	23,143
	6	32,429
	7	34,143
	8	35,143
	9	35,143
	10	46,286
	11	54,429
	12	63,429
